# Mediation Effect of Oxidative Stress on Association Between Selenium Intake and Cognition in American Adults

**DOI:** 10.3390/nu16234163

**Published:** 2024-11-30

**Authors:** Jia-Meng Li, Ya-Zhi Bai, Quan-Ying Liu, Shuang-Qing Zhang

**Affiliations:** National Institute for Nutrition and Health, Chinese Center for Disease Control and Prevention, 27 Nanwei Road, Beijing 100050, China; ljmchinacdc@163.com (J.-M.L.); yzbaichinacdc@163.com (Y.-Z.B.); liuqychinacdc@163.com (Q.-Y.L.)

**Keywords:** selenium intake, cognition, oxidative stress, mediation effect, National Health and Nutrition Examination Survey

## Abstract

Objectives: Dementia affects millions of aged people globally and mainly results from oxidative stress. Selenium shows beneficial effects on dementia however it remains elusive for the mediation effect of oxidative stress on the association between selenium and cognition. The present study firstly investigated the potential mediation role of oxidative stress on the relationship of selenium and cognition. Methods: A total of 2154 adults aged 60 years and older from the National Health and Nutrition Examination Survey 2011–2014 were selected for the study. Weighted multivariate linear regression, weighted logistic regression, and mediation effect analysis were employed to investigate the association among selenium intake, cognition, and oxidative stress. Results: Selenium intake was positively associated with cognition, albumin, and vitamin D, negatively associated with uric acid, and exhibited no correlation with gamma glutamyl transpeptidase (GGT). Cognition was positively correlated with albumin and vitamin D, negatively related to GGT, and had no association with uric acid. Albumin and vitamin D significantly mediated the relationship between selenium intake and cognition, and the mediation proportion values of albumin and vitamin D were 3.85% and 8.02%, respectively. Conclusions: For the first time, our findings demonstrated that higher selenium intake decreased cognitive impairment and oxidative stress levels. Moreover, the relationship between selenium intake and cognition was mediated by oxidative stress.

## 1. Introduction

Dementia, the seventh leading cause of death and one of the major reasons for disability and dependency, affects about 55.2 million people worldwide, and its global cost is projected to grow from $1.3 trillion in 2019 to $1.7 trillion in 2030 [1]. Therefore, it is imperative for the prevention and treatment of dementia. Despite decades of extensive research, effective clinical therapies for dementia remain limited [2]. Fortunately, several studies have indicated that selenium shows beneficial effects on dementia [3].

Selenium, an essential micronutrient for humans provided by various foods, is distributed throughout the body, particularly in the brain [4]. Compared to zinc and copper, selenium attenuated the negative association of co-exposure to arsenic, cadmium, and lead with cognitive function [5]. It plays crucial roles in cognition [1], including antioxidation [6], anti-inflammation [4], modulation of the levels of metals such as zinc and copper [7], enhancement of synaptic plasticity, inactivation of ferroptosis, and regulation of de novo synthesis of L-serine and PI3K/mTOR/GSK3 [4]. Selenium deficiency has been recognized as an important contributing factor to dementia due to its detrimental effects on humans [8]. However, inconsistent results were observed in human studies on the association between selenium and cognition. A cross-sectional study conducted on 1681 Americans over 65 years old found higher cognitive scores in individuals with higher selenium intakes [9]; however, no association was observed between plasma selenium and cognition in 154 Australian adults aged 60 years and older [10]. Therefore, it is necessary to analyze the association between selenium intake and cognition.

Oxidative stress results from the imbalance between reactive oxygen species (ROS) and antioxidant defenses [11]. ROS inflicts damage upon neuronal cellular membranes, proteins, and deoxyribonucleic acid, impairs neuroelectric conduction and synaptic plasticity, and finally leads to dementia [12]. Albumin, gamma glutamyl transpeptidase (GGT), uric acid, and serum vitamin D are usually used as biomarkers of oxidative stress [13]. Although the association between these biomarkers and dietary fat intake had been disclosed [14], the relationship between these biomarkers and trace elements such as selenium has not been investigated. Therefore, these biomarkers are first employed to investigate the relationship between selenium intake and oxidative stress in our study. Mediation effect analysis decomposes the overall impact of exposure on outcomes into a direct effect and an indirect effect through a mediator variable, and its strength lies in the utilization of non-parametric test and the derivation of causality between variables. Some studies found the positive correlation between selenium intake and cognition using multiple linear regression or logistic regression [3,9]. To date, no study analyses the relationship between selenium intake and cognition through mediation effect analysis.

Therefore, for the first time, by virtue of oxidative stress biomarkers and mediation effect analysis, our study aimed to investigate the association between selenium intake and oxidative stress, as well as the association between selenium intake and cognition using the National Health and Nutrition Examination Survey (NHANES) 2011–2014. Our work might provide selenium as a valuable therapy option for the improvement of cognitive function.

## 2. Methods

### 2.1. Study Population

**Figure 1:**
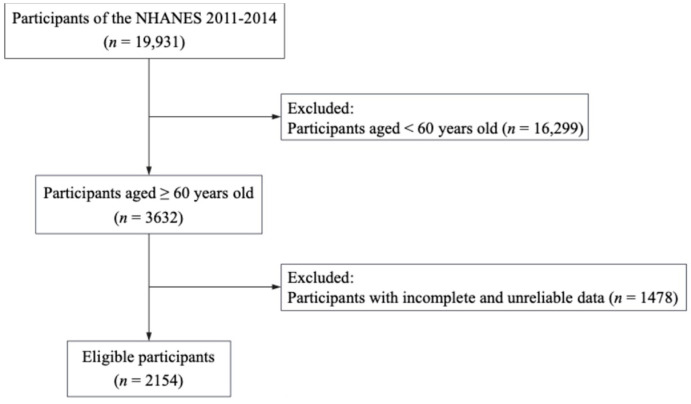
The selection process of the study participants.

The NHANES represents a groundbreaking initiative that aims to evaluate the health and nutritional status of adults and children in the research program. The distinguishing aspect of this survey lies in its comprehensive approach, which integrates interviews and physical examinations. Each year, the survey examines a nationally representative sample comprising approximately 5000 individuals distributed in 15 counties. The National Center for Health Statistics Ethics Review Board approved the present study, and all participants signed informed consent forms. Additional ethics approval was not necessary because our research was based upon a secondary analysis. Our study utilized data from 19,931 people surveyed in the NHANES 2011–2014. Following the exclusion criteria in Figure 1, a total of 2154 participants were eligible for the analysis, in which 745 people took selenium supplementation.

### 2.2. Selenium Intake

Selenium intake was evaluated using two 24 h dietary recalls. Dietary recall interviews were conducted in person by trained diet interviewers fluent in Spanish and English. A Mobile Examination Center (MEC) was utilized to serve areas selected for inclusion in order to provide ease of access to participants. Interviewers that gathered participant information underwent a week-long training program to ensure proper technique and the following of protocol when interviewing participants. The first interview was collected in the MEC in person, and the second one was carried out by telephone 3–10 days later. Upon the completion of the in-person interview, measuring cups, spoons, a ruler, and a food model booklet containing two-dimensional drawings of the various measuring guides available in the MEC were given to participants for use in reporting food amounts during the telephone interview. The total selenium intake was calculated by the average selenium intake of two days of dietary selenium plus supplemental selenium.

### 2.3. Cognitive Function

The cognitive function assessment consisted of the following three parts: (1) the Consortium to Establish a Registry for Alzheimer’s disease (CERAD), (2) the animal fluency test, and (3) the Digit Symbol Substitution test (DSST).

The CERAD word learning subtest was used to assess immediate and delayed recall of new linguistic information (the memory sub-domain) through three consecutive learning trials followed by a delayed recall session. In the learning experiment, participants were instructed to read aloud 10 unrelated words, 1 at a time. Immediately after word introduction, participants recalled as many words as possible. The sequence of 10 words was changed for each of 3 learning trials. The maximum possible score per trial was 10. In the NHANES, the words for the learning trials were displayed in large bold letters on a computer monitor. Participants who were unable to read due to literacy or visual impairment were asked to repeat each word after the interviewer had read it. Delayed word recall occurred after completion of the other two cognitive exercises (animal fluency and DSST) (approximately 8–10 min from the beginning of the word learning trial). The animal fluency test examined categorical language fluency. Participants were asked to name as many animals as possible in one minute. Each named animal will receive one point. In the NHANES, participants were first asked to name three items of clothing, another verbal fluency category, as a practice test. Participants who were unable to name three items of clothing did not proceed with the animal fluency exercise. The DSST is a performance module of the Wechsler Adult Intelligence Scale used to assess subjects’ processing speed, continuous attention, and working memory. Participants taking part in the DSST had two minutes to copy the relevant symbol number adjacent to the 133 boxes. The score was the total number of correct matches. Sample practice tests were conducted before participants began the main test. In the NHANES, participants who were unable to correctly match symbols to numbers on their own during pre-test practice did not continue. The total scores of the three parts were used as the general cognitive function score, the lowest quartile was considered as cognitive impairment, and the rest was normal cognition [15].

### 2.4. Oxidative Stress Biomarkers

Beckman Coulter UniCel DxC800 (Beckman Coulter, Fullerton, CA, USA) used various methods, including a bichromatic digital endpoint method for albumin measurement, an enzymatic rate method to determine GGT, and a timed endpoint method for the measurement of uric acid. Vitamin D, the sum of 25-hydroxyvitamin D2 and 25-hydroxyvitamin D3, was detected by ultra-high performance liquid chromatography-tandem mass spectrometry.

### 2.5. Study Covariates

Covariates were chosen mainly based on factors associated with cognitive function in the previous studies [16,17]. In our study, covariates included age (≥60 years), gender (male and female), race (Mexican American, other Hispanic, Non-Hispanic White, Non-Hispanic Black, and other race), educational level (less than high school, high school, and more than high school), and marital status (married/living with a partner, widowed/divorced/separated, and never married), poverty/income ratio (PIR) (≤1.3, 1.3–4.0, >4.0) [18], and body mass index (BMI) (underweight: <18.5 kg/m^2^; normal: 18.5–24.9 kg/m^2^; overweight: 25–29.9 kg/m^2^; obesity: ≥30 kg/m^2^) [19]. Participants were defined as “drinkers” if they had at least 12 alcohol drinks in the previous year [14]. Smoking at least 100 cigarettes in life was defined as “smokers” [15]. Additionally, the history of diseases (hypertension, diabetes mellitus, coronary heart disease, and stroke) was included [3].

### 2.6. Statistical Analysis

Statistical analysis was conducted using SAS 9.4 (SAS Institute Inc., Cary, NC, USA) and R version 4.3.1. Continuous variables are presented as the mean ± standard deviation (SD) and categorical variables are expressed as cases (n) and percentages (%). Differences among means were analyzed by Rao-Scott χ^2^ test or ANOVA.

With selenium intake as an independent variable and oxidative stress as a dependent variable, weighted multivariate linear regression models were used to explore the association between selenium intake and oxidative stress. Weighted logistic regression models were employed to investigate the relationship between selenium intake and cognition, as well as oxidative stress and cognition. Three models were used in our study. Model 1 was a crude model not adjusted for potential confounding factors. Model 2 was adjusted for age, gender, race, educational level, and marital status. Model 3 was further adjusted for PIR, BMI, smoking, alcohol use, and comorbidities (diabetes, hypertension, stroke, and coronary heart disease).

The strategy of distribution-of-the-product was applied to detect the potential mediation role of oxidative stress in the association between selenium intake and cognitive function by checking (1) the association between selenium intake (the main determinant) and cognition (the outcome), adjusting age, gender, race, educational level, marital status, PIR, BMI, smoking status, drinking status, and diseases history (path c); (2) the association between selenium (the main determinant) and oxidative stress (the outcome), adjusting age, gender, race, educational level, marital status, PIR, BMI, smoking status, drinking status, and diseases history (path a); (3) the effect of selenium (the main determinant) on cognition (the outcome), making adjustment for the same covariates (path c’); and (4) the indirect effect (path a × path b). The R mediation package was used to evaluate the mediation effect of oxidative stress on the relationship between selenium intake and cognition. Mediation proportions and *p*-values were obtained after 1000 simulations using the quasi-Bayesian Monte Carlo method [20]. All statistical tests were two-sided, and *p* < 0.05 was regarded as statistically significant.

## 3. Results

### 3.1. General Characteristics of Participants

The characteristics of individuals categorized by cognition status are summarized in Table 1. There were significant differences between subjects with cognitive impairment and normal cognition in age, race, educational level, marital status, drinking, PIR, diabetes mellitus history, hypertension history, stroke history, and oxidative stress biomarkers (*p* < 0.05) but not in gender, smoking status, BMI, and coronary heart disease history (*p* > 0.05). In addition, selenium intakes in the normal cognition group were higher than those in the cognitive impairment group (*p* < 0.01).

### 3.2. Relationship Among Selenium Intake, Cognition, and Oxidative Stress

The relationship between selenium intake and cognition is presented in Table 2. The results of weighted logistic regression models showed that there was a statistically significant positive association between selenium intake and cognition in models 1, 2, and 3 (*p* < 0.0001).

The relationship between oxidative stress and cognition is displayed in Table 3. The results of weighted logistic regression models showed that GGT was negatively correlated with cognition in all models (*p* < 0.05). Albumin was positively correlated with cognition in model 1 (*p* = 0.0011) and model 3 (*p* = 0.0154), and was negatively associated with cognition in model 2 (*p* = 0.0084). In all models, vitamin D was positively associated with cognition (*p* < 0.05). The association between uric acid and cognition was not found in all models (*p* > 0.05).

The relationship between selenium intake and oxidative stress is exhibited in Table 4. The results of weighted multivariate linear regression models indicated that selenium intake was positively correlated with albumin and vitamin D (*p* < 0.05), and was negatively correlated with uric acid in all models (*p* < 0.05). No significant association was found between selenium intake and GGT in all models (*p* > 0.05).

### 3.3. Mediation Effect of Oxidative Stress on Association Between Selenium Intake and Cognition

**Figure 2:**
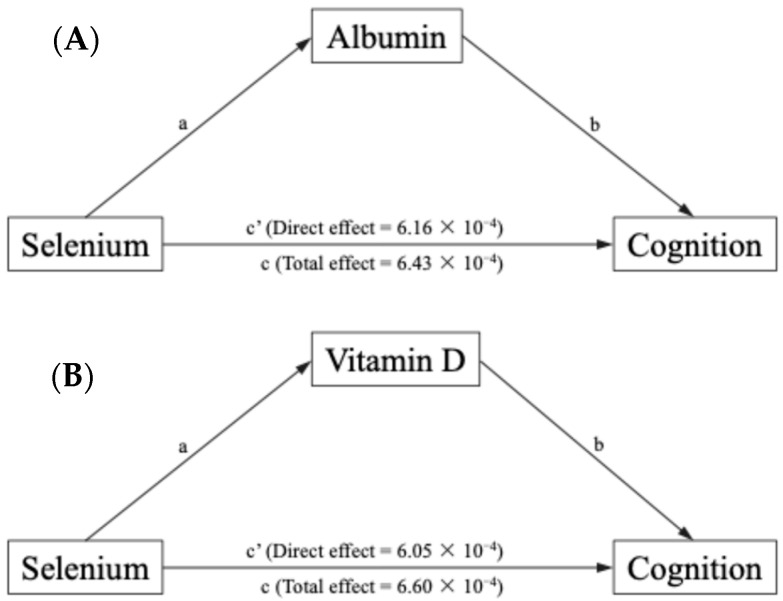
Indirect effects of selenium on cognition in mediation design. (**A**) The mediation effect of albumin on the association between selenium and cognition. (**B**) The mediation effect of vitamin D on the association between selenium and cognition.

Based on the analysis among selenium intake, cognition, and oxidative stress, significant associations were chosen and the mediation was tested. Table 5 showed that after adjusting the controlled variables, albumin significantly mediated the link between selenium intake and cognition (proportion mediated, 3.85%; indirect effect, 2.61 × 10^−4^ [6.30 × 10^−6^–5.31 × 10^−4^], *p* < 0.01), and the direct effect of selenium intake on cognition in the absence of the mediation of albumin was also significant (*p* < 0.01). The relationship between selenium intake and cognition was significantly mediated by vitamin D (proportion mediated, 8.02%; indirect effect, 5.36 × 10^−5^ [1.63 × 10^−5^–9.90 × 10^−5^], *p* < 0.01), as shown in Figure 2.

## 4. Discussion

Our study investigated the relationship between selenium intake and cognition, and the results showed that subjects with higher selenium intake had a lower risk of cognitive impairment, which aligned with the previous reports [3,9,16,21]. However, a randomized clinical trial reported no relationship between selenium intake and cognition, and the discrepancy may be ascribed to only male subjects involved in the study [22].

Our results also disclosed that oxidative stress was a risk factor for cognitive impairment. Albumin was positively correlated with cognition in our research, which was consistent with the previous study [13,23,24]. Due to its distinct biological structure, specifically the cysteine residues at position 34, albumin had the potential to alleviate excessive oxidative stress caused by inflammation in aging neuronal cells, so it was regarded as an important biomarker for the evaluation of cognitive impairment in aging brains [23,25]. Participants with a higher vitamin D level exhibited better cognitive performance, which was in line with the previous results [26,27]. Vitamin D played a fundamental role in the maintenance of normal physiological function of neurons through the regulation of neuroprotective factors and/or oxidative stress [28]. Participants with a lower GGT level had higher cognition, which was in agreement with the previous reports [13,14,29]. GGT served a crucial function in maintaining the transport of glutathione into cells, thereby favoring cellular protective antioxidant capability [30]. Although there were similar uric acid levels in our research (5.6 mg/dL) and previous reports (5.3 [31] and 5.2 [32] mg/dL), the level of uric acid in our investigation was not correlated with cognitive performance, which was inconsistent with previous cross-sectional studies [31,32], possibly due to the Chinese population that participated in their reports. The inconsistency reminded us that the characteristics of the study population should be fully considered. If the study involved people from different regions, it was necessary to conduct an in-depth analysis of the various characteristics of the population. Oxidative stress level was significantly increased in the brain tissues of patients with cognitive impairment [33,34], and our results strongly supported the negative association between oxidative stress and cognition.

For the first time, we discovered that selenium intake was negatively associated with oxidative stress. Selenium was a key factor in maintaining human health by clearing free radicals, improving the activity of antioxidant enzymes, and inhibiting oxidative stress reactions [35,36]. Additionally, our present study firstly proposed that albumin and vitamin D had an incomplete mediated effect (i.e., the indirect effect was small and the direct effect was significant) on the association between selenium intake and cognition, suggesting that additional pathways (such as anti-inflammation and ferroptosis) besides antioxidation could be involved in the regulation of effects of selenium on cognition [4,37].

This study had several strengths. Firstly, it was the first study to investigate the relationship between selenium intake and cognition mediated by oxidative stress. Secondly, large national representative samples of elderly adults in the US increased the extrapolation of the results. Thirdly, all information were derived from highly valid NHANES records, and potential confounding factors were controlled in data analysis. However, several limitations also existed in this study. First, the NHANES was a cross-sectional study, which did not allow causal inferences. Second, dietary selenium data were self-reported, which might be subject to recall bias. Third, socioeconomic status and lifestyle factors may have important impacts on selenium intake and cognitive function. People with higher socioeconomic status and/or healthy lifestyles have opportunities to consume more selenium.

## 5. Conclusions

For the first time, our findings demonstrated that higher selenium intake decreased cognitive impairment and oxidative stress levels. Moreover, the relationship between selenium intake and cognition was mediated by oxidative stress.

## Figures and Tables

**Table 1:** General characteristics of participants (*n* = 2154).

Characteristic	Cognitive Impairment		*p*
	Yes (*n* = 544)	No (*n* = 1610)	
Age, years ^a^	73.57 ± 6.43	68.03 ± 6.31	<0.01
Gender ^b^			0.80
Male	302 (47.77)	741 (46.95)	-
Female	242 (52.23)	869 (53.05)	-
Race ^b^			<0.01
Mexican American	63 (7.19)	114 (2.58)	-
Other Hispanic	84 (9.92)	114 (2.52)	-
Non-Hispanic White	202 (60.95)	922 (83.74)	-
Non-Hispanic Black	172 (17.87)	310 (6.14)	-
Other Race	23 (4.08)	150 (5.03)	-
Education ^b^			<0.01
Less than high school	259 (37.66)	219 (9.96)	-
High school	142 (30.43)	376 (20.65)	-
More than high school	143 (31.90)	1015 (69.39)	-
Marital status ^b^			<0.01
Married or living with a partner	279 (55.90)	998 (69.14)	-
Widowed/separated/divorced	237 (40.19)	520 (27.08)	-
Never married	28 (3.91)	92 (3.79)	-
Smoker ^b^			0.78
Yes	286 (50.16)	808 (49.12)	-
No	258 (49.84)	802 (50.88)	-
Drinker ^b^			<0.01
Yes	347 (61.72)	1156 (75.73)	-
No	197 (38.28)	454 (24.27)	-
Income ^b^			<0.01
PIR ≤ 1.3	258 (38.16)	335 (12.44)	-
PIR 1.3–4.0	225 (46.81)	734 (43.52)	-
PIR > 4.0	61 (15.03)	541 (44.04)	-
BMI, kg/m^2 b^			0.10
Underweight: <18.5	13 (3.79)	20 (1.16)	-
Normal: 18.5–24.9	136 (25.39)	402 (25.62)	-
Overweight: 25–29.9	186 (35.94)	572 (35.78)	-
Obesity: ≥30	209 (34.87)	616 (37.43)	-
Diabetes mellitus ^b^			<0.01
Yes	194 (32.54)	403 (20.06)	-
No	350 (67.46)	1207 (79.94)	-
Hypertension ^b^			<0.01
Yes	382 (69.31)	963 (56.79)	-
No	162 (30.69)	647 (43.21)	-
Stroke ^b^			<0.01
Yes	61 (10.68)	85 (4.97)	-
No	483 (89.32)	1525 (95.03)	-
Coronary heart disease ^b^			0.05
Yes	68 (12.97)	138 (9.06)	-
No	476 (87.03)	1472 (90.94)	-
Dietary + Supplemental selenium intake, μg/d ^a^	113.09 ± 53.80	134.67 ± 63.29	<0.01
GGT, U/L ^a^	28.99 ± 42.51	23.75 ± 22.10	<0.01
Albumin, mg/mL ^a^	4.12 ± 0.35	4.22 ± 0.27	<0.01
Uric acid, mg/dL ^a^	5.68 ± 1.44	5.57 ± 1.44	<0.01
Vitamin D, nmol/L ^a^	78.35 ± 32.81	84.30 ± 32.48	<0.01

^a^ Continuous variables with a normal distribution were expressed as mean ± SD and *p* value was tested by ANOVA. ^b^ Categorical variables were expressed as the number of subjects (percentage) and *p* value was tested by the Rao-Scott χ^2^ test. BMI: body mass index; PIR: poverty/income ratio; GGT: gamma glutamyl transpeptidase. “-“ means blank.

**Table 2:** The relationship between selenium and cognition (*n* = 2154).

Variables	OR (95% CI)	*p*
Model 1	1.007 (1.004, 1.010)	<0.0001
Model 2	1.005 (1.002, 1.008)	0.0046
Model 3	1.004 (1.001, 1.007)	0.0191

Model 1: Unadjusted. Model 2: Adjusted for age, gender, race, marital status, and educational level. Model 3: Adjusted model 2 variables and family poverty/income ratio, body mass index, smoking, alcohol use and comorbidities (diabetes, hypertension, stroke, and coronary heart disease). CI: confidence interval; OR: odds ratio.

**Table 3:** The relationship between oxidative stress and cognition (*n* = 2154).

Variables	GGT		Albumin		Uric Acid		Vitamin D	
	OR (95% CI)	*p*	OR (95% CI)	*p*	OR (95% CI)	*p*	OR (95% CI)	*p*
Model 1	0.994 (0.990, 0.998)	<0.001	3.067 (1.621, 5.804)	0.001	0.952 (0.866, 1.046)	0.297	1.006 (1.002, 1.010)	0.005
Model 2	0.993 (0.988, 0.998)	0.014	0.854 (0.820, 0.889)	0.008	1.057 (0.941, 1.187)	0.339	1.006 (1.003, 1.010)	<0.001
Model 3	0.994 (0.988, 0.999)	0.030	2.073 (1.161, 3.701)	0.015	0.857 (0.823, 0.893)	0.236	1.005 (1.002, 1.009)	0.007

Model 1: Unadjusted. Model 2: Adjusted age, gender, race, marital status, and educational level. Model 3: Adjusted model 2 variables and family poverty/income ratio, body mass index, smoking, alcohol use, and comorbidities (diabetes, hypertension, stroke, and coronary heart disease). CI: confidence interval; GGT: gamma glutamyl transpeptidase.

**Table 4:** The relationship between selenium and oxidative stress (*n* = 2154).

Variables	Model 1		Model 2		Model 3	
	*β* (95% CI)	*p*	*β* (95% CI)	*p*	*β* (95% CI)	*p*
GGT	−0.0088 (−0.031, 0.013)	0.4153	−0.0145 (−0.0385, 0.0095)	0.2270	−0.0125 (−0.0385, 0.0134)	0.3319
Albumin	0.0005 (0.0003, 0.0008)	<0.0001	0.0004 (0.0002, 0.0006)	0.0019	0.0003 (0.0001, 0.0005)	0.0063
Uric acid	−0.0001 (−0.0015, 0.0014)	0.9272	−0.0018 (−0.0033, −0.0004)	0.0145	−0.0016 (−0.0030, −0.0002)	0.0246
Vitamin D	0.0129 (−0.0282, 0.0539)	0.5280	0.0356 (0.0001, 0.0710)	0.0491	0.0308 (−0.0002, 0.0618)	0.0514

Model 1: Unadjusted. Model 2: Adjusted age, gender, race, marital status, and educational level. Model 3: Adjusted model 2 variables and family poverty/income ratio, body mass index, smoking, alcohol use, and comorbidities (diabetes, hypertension, stroke, and coronary heart disease). CI: confidence interval; GGT: gamma glutamyl transpeptidase.

**Table 5:** The mediation effect of oxidative stress on the association between selenium and cognition (*n* = 2154).

Variables	Estimate	95% CI		*p*
		Lower	Upper	
Albumin				
Total effect	6.43 × 10^−4^	3.70 × 10^−4^	9.15 × 10^−4^	<0.01
Direct effect	6.16 × 10^−4^	3.45 × 10^−4^	8.81 × 10^−4^	<0.01
Indirect effect	2.61 × 10^−4^	6.30 × 10^−6^	5.31 × 10^−4^	<0.01
Proportion mediated	3.85 × 10^−2^	9.27 × 10^−3^	9.30 × 10^−2^	<0.01
Vitamin D				
Total effect	6.60 × 10^−4^	3.90 × 10^−4^	9.22 × 10^−4^	<0.01
Direct effect	6.05 × 10^−4^	3.40 × 10^−4^	8.69 × 10^−4^	<0.01
Indirect effect	5.36 × 10^−5^	1.63 × 10^−5^	9.90 × 10^−5^	<0.01
Proportion mediated	8.02 × 10^−2^	2.35 × 10^−2^	17.53 × 10^−2^	<0.01

CI: confidence interval.

## Data Availability

Datasets are publicly available at https://www.cdc.gov/nchs/nhanes/index.htm (accessed in 2017). The data have not been previously presented orally or by poster at scientific meetings.

## Abbreviations

BMI	body mass index
CERAD	Consortium to Establish a Registry for Alzheimer’s disease
DSST	Digit Symbol Substitution test
GGT	gamma glutamyl transpeptidase
NHANES	National Health and Nutrition Examination Survey
PIR	poverty/income ratio
ROS	reactive oxygen species
